# Editorial: Epigenetics of metabolism, immunology and aging

**DOI:** 10.3389/fgene.2023.1135889

**Published:** 2023-01-18

**Authors:** Hailuan Zeng, Xiaofan Lu, Heng Sun, Haitao Wang

**Affiliations:** ^1^ Department of Endocrinology and Metabolism, Zhongshan Hospital, Fudan University, Shanghai, China; ^2^ Human Phenome Institute, Fudan University, Shanghai, China; ^3^ Department of Cancer and Functional Genomics, Institute of Genetics and Molecular and Cellular Biology, CNRS/INSERM/UNISTRA, Illkirch, France; ^4^ State Key Laboratory of Natural Medicines, Research Center of Biostatistics and Computational Pharmacy, China Pharmaceutical University, Nanjing, China; ^5^ Cancer Center, Faculty of Health Sciences, University of Macau, Macau, Macau SAR, China; ^6^ MOE Frontiers Science Center for Precision Oncology, University of Macau, Macau, Macau SAR, China; ^7^ Zhuhai Research Institute, University of Macau, Zhuhai, China; ^8^ Thoracic Surgery Branch, Center for Cancer Research, National Cancer Institute (NCI), Bethesda, MD, United States

**Keywords:** methylation, histone modification, metabolism, immune, epigenetic age, cancer

The incapacitation and dysfunction of metabolism and immunity are always coupled with aging, contributing to the development of a serial of aging-related diseases, like cancer. Previous studies indicated that many genetic and environmental factors are involved in aging-related diseases. Notably, these diseases are also accompanied by epigenetic changes in DNA methylation, post-translational modifications of histones, RNA-mediated processes, transcription factor binding, and high-order chromatin organization (Booth and Brunet 2016). Besides, increasing evidence has shown that the dysregulation of epigenetic pathways is crucial for coordinating metabolic reprogramming and cellular immunological events in response to extracellular stimuli, leading to and/or fueling the aging-related diseases (Cao and Yan 2020; Sun et al., 2022). Hence, in this Research Topic, we focus on the most recent advances that help understand the roles of epigenetics in aging, metabolism, immunology, and cancer.

The correlation of DNA CpG methylation and aging has been well studied, which serves as the basis for establishment of biological age molecular estimators, such as Horvath’s pan-tissue clock and Hannum’s clock (Horvath and Raj 2018; Noroozi et al., 2021). These epigenetic clocks might be affected by environmental factors, lifestyles, psychological states, and medical treatments (Noroozi et al., 2021). In the current Research Topic, Li et al. performed a genome-wide methylation study to explore the effect of metformin, a widely used drug for diabetes mellitus, on the epigenetic age of peripheral blood in patients with diabetes mellitus, and they found a strong association between metformin intake and slower epigenetic aging by Horvath’s clock and Hannum’s clock. In an epigenome-wide association study on biomarkers of liver function in 960 HIV-infected men, Titanji et al. identified nine DNA methylation sites annotating to *TMEM49*, *SOCS3*, *FKBP5*, *ZEB2*, and *SAMD14* genes that were positively associated with serum albumin, and reported that a higher PhenoAge score was significantly associated with a lower level of serum albumin. Moreover, by combining DNA methylation and transcriptome analysis, Wu et al. identified seven key genes that affect the occurrence and development of obesity by influencing the immune microenvironment of adipose tissue. Further, a connective map analysis of drugs suggested that four small-molecule drugs might have therapeutic effects on obesity. The study suggests that epigenetic factors could mediate the interaction between metabolism and immunity, thus shedding light on identifying novel drugs targeting obesity.

In addition to DNA methylation, other epigenetic mechanisms and technologies have been reported and used in clinical applications. For example, long non-coding RNAs have been shown to regulate gene expressions through recruiting, repelling, or affecting the expression of DNA methyltransferases and TET enzymes (Huang et al., 2022). In this Research Topic, Wang et al. performed a blood circRNA-seq analysis on Chinese participants and identified 5 age-related biomarkers that can be used to develop forensic age prediction models with a mean average error less than 10 years. Zhang et al. developed a risk model based on 11 m6A-related long non-coding RNAs for predicting gastric cancer prognosis and sensitivity to immune checkpoint inhibitors. Gong et al. identified eight N7-Methylguanosine (m7G)-related prognostic genes that can be used to categorize sepsis patients into two molecular subtypes with different metabolic activities and immune status. Wang et al. showed that the expression of CD146, a gene correlated with m5C RNA methylation modification, is linked to poor prognosis in osteosarcoma and can be used to predict response to immunotherapy. Zhao et al. evaluated the activation status of the histone modification pathway in cervical cancer and develop a prognostic model to predict clinical outcomes. These studies may provide valuable insights and potential applications for personalized treatment strategies for heterogeneous diseases.

This Research Topic also includes reviews on different aspects of epigenetic regulations in signaling pathways and pathological processes. For example, Li et al. reviewed the regulatory mechanisms of N6-methyladenosine RNA methylation, the techniques for detecting N6-methyladenosine methylation, the role of N6-methyladenosine modification in cancer and other diseases, and the potential clinical applications. Yin et al. introduced the role of non-proteolytic ubiquitination in tumorigenesis and related signaling pathways. Sun et al. provided an overview of the role of rapamycin (TOR) signaling pathway regulator (TIPRL) in cancer development, as well as the TIPRL/protein phosphatase 2A (PP2A) axis and its epigenetic regulation. In addition, Cai et al. summarized the interactions of genetics, environmental factors, and epigenetics on knee osteoarthritis, with a focus on DNA methylation, histone modification, and non-coding RNA.

Epigenetic changes associated with the onset and progression of aging-related diseases could serve as biomarkers for the diagnosis and prognosis of these diseases. In this Research Topic, several studies have investigated the role of fatty acid metabolism genes (Nie et al.), glycometabolism (Yu et al.), and genetic variants (Zhao et al.) on the prognosis and response to immunotherapy in various types of diseases, including clear cell renal cell carcinoma (ccRCC), hepatocellular carcinoma (HCC), and uveal melanoma (Liang et al.). These studies have shown promising results in using multidisciplinary data analysis to better understand the relationship between epigenetics and diseases, and have the potential to lead to the development of more effective treatments for these conditions. In particular, the findings on the epigenetic regulation of metabolism, immunology, and cancer may improve our understanding of the underlying causes of these diseases and potentially improve health and longevity in individuals.
